# Observations on the Epidemic Yellow Fever of Natchez, and of the South-west

**Published:** 1842-03

**Authors:** John W. Monette

**Affiliations:** Washington, Mississippi


### WESTERN JOURNAL

OF

### MEDICINE AND SURGERY.

---

MARCH, 1842.

---

ART. I.—*Observations on the epidemic Yellow Fever of Natchez, and of the South-west.* By JOHN W. MONETTE, M. D., of Washington, Mississippi.

(*Continued.*)

### THEORY OF YELLOW FEVER MIASM.

To produce yellow fever as an epidemic in our cities, we conceived three circumstances or grades of action necessary:

1. *Miasm*, or the simple basis, which uncombined we think harmless.

2. The union of miasm with impure air, or with air which has been exhausted by respiration, and charged with human effluvia. This combination we term *malaria* or *infectious air*. This is the proper nidus or receptacle for the dissemination and extension of personal infection; and is *always requisite* for the spread of this disease as an epidemic.

3. *Infection*, or the union of personal infection with *malaria* or infectious air. In this state the poison becomes active, and constitutes the efficient, predisposing, and exciting cause of epidemic yellow fever. It constitutes an *aerial poisonous ferment*, extremely subtile in its nature and properties.

These views we will endeavor to illustrate as we progress. The term *miasm*, as an agent in the production of yellow fever, we use in a sense somewhat different from its common acceptation, and, as we think, with equal propriety and reason.

I. *The Miasm of Yellow Fever.*—This we believe a subtile, gaseous, invisible and inodorous matter, generated by the action of the sun, or by solar heat, upon *common atmosphere*, independently of any effluvia, fetor, or exhalation from the decomposition of animal matters, or of any exhalation from marshes, dry earth, or vegito-animal compounds, or any of the commonly received sources of miasm. We believe it the result of some unknown combination of the solar rays with portions of the atmosphere, in certain situations, and under certain circumstances. These circumstances are, that the air shall be to a certain extent confined or stagnant; such as we have in cities in sultry oppressive weather, when the temperature in the shade is steadily above 90° of Fahrenheit during some portion of the day, or above 125° in a fair exposure to the open rays of the sun. The precise nature of this combination we are unable to explain satisfactorily. The doctrine of marsh-miasm conceives something generated by a similar process. We suppose this miasm to be perfectly free from morbific properties in its *simple state*, but that it becomes morbific by combination with impure air; that by its specific gravity it becomes particularly separated from common air, and settles near the surface of the ground, and in low, damp, confined places, similar to collections of carbonic acid gas; that it is entirely neutralised by a temperature of 45°, and is partially neutralised by a temperature as high as 55°; that it generates slowly, and only while the temperature is above 90° in the shade.

Hence we infer that it can accumulate only after several days of continued high temperature, during a period, and in situations where the extreme heat by day in the shade is above 90°, and the lowest temperature at night is above 55°. We suppose, judging from all the circumstances of weather, preceding all the epidemics of this kind in the United States, that it will not accumulate in prejudicial quantities, in less than 10 or 12 days of unremitted hot, calm, and sultry weather, such as we have described; that whatever amount may have accumulated in any number of successive hot days, will be destroyed or neutralised by one cold night, with the mercury at any time below 55°; or a strong wind, or a tornado, would disperse or waft it off. Hot weather favors its generation, and calm weather its accumulation.

We believe it requires a temperature of 90° in the shade for its generation, because the yellow fever has never spread as an epidemic in any place where the mercury of Fahrenheit by day had not ranged up to 90°, for at least 10 days previous to the epidemic, with a night temperature above 60° in the coldest exposure. We infer that one night, in which the mercury would sink to 55°, would destroy all that had accumulated in a week.

The *mean* temperature, as generally estimated, is no test—*no criterion*, but the *actual highest range of the mercury by day and by night*. We infer that the miasm once accumulated is not injured by damp, or foggy air, unless the temperature fall below 80° by day and 65° by night. If several days of damp, foggy, or misty weather, with high temperature, succeed a week of hot, calm, and sultry weather, the miasm previously generated remains in *statu quo*. Low damp situations in cities attract and retain it.

This miasm is seldom generated in our latitude, except in cities and towns, where the temperature in summer is always at least 3° or 4° above the temperature in the country; and this increased temperature is produced by the great amount of reflecting and radiating bodies in those places.

The miasm accumulates in the West Indies, more or less during at least six months in the year, because the temperature during that time is always above the point requisite for its neutralisation; and for more than nine months in the year, the temperature is not reduced to the point requisite to destroy or neutralize *infection*, or to 32°.

We infer this miasm is innocuous in a pure and often renewed atmosphere, because we find that in many cities and towns, when there has been free ventilation, there has been no epidemic yellow fever, although the mercury has ranged as high as 90° or 93° for several days together; but under the same temperature, with a stagnant atmosphere, constituting sultry weather, there has been yellow fever in its epidemic form. Stagnant atmosphere in a city becomes rapidly contaminated, where probably 100 or even 500 persons to every acre of surface are each incessantly respiring the air, besides the constant exhalations from their bodies. By these means alone, the mass of atmosphere in a city would become perfectly saturated with these exhalations in the space of a few days of sultry weather, besides being exhausted of its oxygen or vitalizing principle, whatever it be. The miasm uniting with this kind of atmosphere, becomes slightly morbific, and predisposes to yellow fever—*but not to bilious fever*, as we shall show hereafte r.

In this state it becomes *infectious air*, or *malaria;* and this air, confined in a high temperature, and combined with the morbid effluvia from a patient laboring under yellow fever, after a few days acquires active morbific properties, which will produce malignant yellow fever in those who are unacclimated to such air.

When the air of a city is prepared for this morbific combination, the introduction of a few cases of yellow fever, or beds, and large quantities of porous and woollen goods from an infected part of the city, will greatly increase the danger of an epidemic yellow fever in such place.

II. *Malaria or infectious air.*—We have said that this union of miasm with the contaminated air of cities in hot sultry weather, predisposes to yellow fever; and the longer the duration of such weather the stronger will be the predisposition, and *cæteris paribus*, stronger in *strangers* than in resident or acclimated persons.

The whole population of the city or town may labor under this predisposition, and yet remain healthy and unconscious of danger; and if nothing occur as *exciting causes*, they may escape an epidemic, or even sporadic cases. But on the contrary, some cases will be excited into action by extraordinary circumstances of fatigue, exposure, or by some imprudence.

Every case, *however excited into action*, sporadic or otherwise, increases the danger and the predisposition, in those immediately about the sick. At such times, the presence of a large number of *northern strangers* in the city is one of the most dangerous circumstances, and tends more than any thing else to lead on the epidemic: because the atmospheric predisposition, which would be innocent to the resident population, would excite sporadic cases in the strangers; and every sporadic case at such time increases the virulence of the local malaria, and thus hastens on the epidemic. Each patient with yellow fever at that time, throws off into the local atmosphere of his room, an effluvium, a subtile gaseous emanation from his body, which tends strongly to render that atmosphere morbific; and if that room be closed for several days afterwards, with a steady temperature between 85° and 90° of Fahrenheit, the air in it will become infected, and will produce yellow fever in some of those who may enter and breathe it only for a few minutes, and especially in such as are not acclimated. When the general atmosphere in the vicinity is becoming assimilated, strangers and unacclimated persons will be among the first, if not the very first, victims.

But, at such time of general predisposition, if steamboats, charged with infected air from an infected port, and freighted with goods and passengers from the infected district, arrive daily at the wharves, and discharge those goods and passengers who have already imbibed the disease; and are also visited by scores of the idle and curious, besides those whose duty requires them to be on board, we shall soon see what are called sporadic cases springing up in different parts of the city, and chiefly, too, in those who *have been* in the vicinity of the wharves or *on board* the steamboats. Thus the epidemic yellow fever is always ushered in, not only at Natchez, but at all other points where it occurs on the lower Mississippi.

Hence, we conceive after miasm is formed, the *first stage* of contamination is produced in the general air of a city by the respiration, and the exhalations from human bodies *in health*; the *second stage*, by the emanations from the same laboring under this peculiar disease; and this constitutes *infection*, which is a virulent atmospheric poison.

III. *Infection.*—When the local atmosphere of a city, town, or even a house, has gone through all the previous stages, it possesses very virulent qualities, and assumes properties which are peculiar to it in this state. It then has become infection; and in addition to its morbific qualities, it has the property or power of extending itself in an impure atmosphere, or of assimilating to itself a certain portion of the contaminated atmosphere around. Portions of this infected air may be transported from one place to another, and then introduced into contaminated air, it will assimilate a circumscribed portion of it, until the latter will possess the same morbific properties as that which was introduced. This process we conceive to be a "gaseous ferment." Dr. Cartwright calls it the "assimilating process."*

We call it a gaseous ferment from the analogy which appears to exist between its operations and those of the common carbonic acid gas fermentation: like it this infection requires a certain basis for its action; it requires a certain degree of temperature for a certain time, with quietude. In both a certain process, the fermentative, will at length be completed; but when this process is complete, a small portion of the product, introduced into a similar basis, and in similar temperature, will excite the process of fermentation or assimilation much

*Medical Recorder, Vol. ix. pp. 9 and 10.

more speedily, and probably at a lower temperature than was required in the first process without the *leaven.* In all these particulars, as well as others, the analogy holds good.

Without the proper basis for the action of the assimilating process, the infection ceases to extend itself; thus it cannot extend the sphere of its virus without contaminated air, or through a pure atmosphere; for, as Dr. Paloni of Naples observes, relative to the yellow fever of Leghorn, which desolated that city in the autumn of 1804, "the infection of this fever is of such a constitution, that *pure and renewed air decomposes its fomes,* at a small distance from the sick; on the other hand, *air that stagnates, and is replete with animal exhalations, easily becomes a vehicle for it.* Hence it happens, that as soon as the disorder broke out, we saw it rage most fiercely in the *most filthy and least ventilated* parts of the city. *Pure, fresh, renewed air, destroys its infection.*"*

This is precisely what we contend for, viz: that yellow fever is *not infectious in a pure, free air,* and that it *is infectious or communicable in a contaminated atmosphere.*

We have already alluded to the diverse and opposite sources of yellow-fever miasm, as adduced by the advocates of its domestic origin in contradistinction to its introduction from infected ports or by infected vessels. There are scarcely any two ports or towns where it has been ascribed to the same combination of local causes. In every place where it has prevailed the source has been peculiar; no two epidemics have been produced by the same local causes, if the theories of gentlemen be correct. All the speculations and theories that have been adduced in favor of its local origin in any port or town in the United States, have had an advocate in Natchez or New Orleans; none having been too weak or too absurd to find a friend or admirer here.

We ascribe the epidemic yellow fever of Natchez and all our south-western towns, to one and the same cause, or to the same combination of circumstances, which are as follows:

---

* Medical Repository, Vol. viii. pp. 426—7, &c.

1. Hot, dry, and sultry weather during August and September; but especially for 15 or 20 days in succession, immediately preceding the epidemic visitation; during which period the mercury of Fahrenheit shall range regularly as high as 90° by day, and above 70° at night, with that peculiar feeling of the atmosphere which is generally termed *oppressive*—or as Dr Cartwright designates it, a "breathless state of the atmosphere."*

2. The existence of yellow fever as an epidemic in New Orleans, at least 10 days before it appears in Natchez.

3. Regular, daily and uninterrupted steamboat communication with New Orleans.

4. A general state of salubrity in the city up to the outbreak of the disease, and the same state of salubrity through the country generally, both before and during the epidemic.

5. Public attention diverted to imaginary causes in the city, and apprehension quieted by a reliance upon the security gained by the removal of certain imaginary sources, and the prophylactic virtues of lime; while the real danger of importation is disregarded. These we believe to be the most invariable circumstances which precede epidemic yellow fever in Natchez; and Natchez was a stranger to these visitations previously to the autumn of 1817, which was soon after the introduction of steamboats on the Mississippi river.

As this was the first time that Yellow Fever was known as an epidemic in Natchez, I will give a short notice of it, for which I am indebted to Dr. Perlee, one of the most able physicians of the city for several years.†

*Epidemic of* 1817.—Occasional cases were seen in Natchez as early as August preceding the epidemic; but where they contracted the disease I have not been able to learn. The summer had been very hot, with occasional showers up to the first of September, with a sultry condition of the atmosphere afterwards. Dr. Perlee says, "the weather had been such as

---

* See Medical Recorder, Vol. ix, &c.

† See Chapman's Medical Journal, Vol. iii. p. 6.

*predisposed* very strongly to violent disease, even early in September, and required only a small concentration of power to produce a sweeping epidemic." There had been no uncommon sickness, except this "strong predisposition to violent disease," which no doubt the Doctor thought he perceived. "At this time, when the population was *highly susceptible*, the Washington *steamboat* reached us from New Orleans, with persons on board, ill of yellow fever, some of whom were landed; and several young men from town went on board, who were all taken sick soon after and died. *The disease spread rapidly and with most destructive malignity.* For some time it had its sway over the whole city. On the 28th of September the physicians publicly announced the existence of yellow fever, and a large portion of the population retired to the country." It continued to prevail with slight interruptions until the 9th of November; and several cases occurred six days later in those who returned after frost to their houses which had been closed during their absence.

The whole numberof deaths in the city, during this epidemic, was one hundred and thirty-four; besides some in the country who contracted their disease in the city.

The "predisposing causes" to which Dr. Perlee ascribed this epidemic, are the exhalations from stagnant pools, from city filth, from newly made ground in levelling the streets, and from an old grave-yard near the middle of the city. This heterogeneous mixture of effluvia, he supposed, produced the high state of "general predisposition," and prepared the inhabitants for an epidemic; and required only the aid of some *exciting* cause to produce a "sweeping epidemic." Although he was an advocate for the local origin of yellow fever, and would not ascribe it under any circumstances to importation, he leaves us to infer that the Washington steamboat with her yellow-fever patients, and her infected cabin, was the exciting cause which alone was wanting up to that time. In this we cordially agree with him, and believe that the same degree of predisposition, without similar "exciting causes," would every summer pass off without an epidemic. Although Dr.

Perlee was an advocate for the local origin of this disease, he evidently considered the arrival of the steamboat Washington as an important incident in the history of this epidemic. At that time not more than a half dozen steamboats were on the Mississippi, and the arrival of this boat from New Orleans was a matter of much interest and curiosity; so that many went on board to gratify their curiosity, and thus aided in introducing the disease into the city.

*The epidemic of* 1819 was the next visitation. It began on the 4th of September by a few scattering cases, and was not considered epidemic until the 14th of September. It continued to prevail with great mortality until the first of December, when 200 persons had fallen victims to its fury.

Dr. Perlee ascribes this epidemic to the atmospheric predisposition generated by similar causes to those enumerated in the last epidemic. At this time steamboat arrivals were so common that he does not refer to that as an exciting cause.

In 1819, the yellow fever raged with great violence in New Orleans, from and after the middle of August. Cases had occurred occasionally on board the shipping and about the wharves as early as June. Toward the middle of August it raged severely among the shipping and spread to the adjacent streets, and also to the boats which had descended the river. *Not a ship escaped* without the loss of one or two hands, and some lost their whole crews; many steamboats became infected, and lost many of their passengers and crews. It was not entirely destroyed until January following.*

During the years 1820, 1821, and 1822, Natchez was exempt from yellow fever epidemics; yet the writer is well convinced that during each of these years, and especially in 1822, there were several deaths from this disease in the city which were traced to infection from New Orleans introduced in bales of blankets and other woollen goods, and some cases in persons who visited New Orleans on business, and were attacked after their return.

---

* Medical Repository, New Series, Vol. vi: pp. 6—20.

*The epidemic of* 1823.—This was probably the most terrific that has ever visited Natchez, or any other city of its population. The first cases occurred between the 12th and 20th of August. It continued to rage with great mortality until checked by frost near the first of November. By that time about 320 souls had died from this disease.

The weather for some weeks preceding this epidemic was very warm and oppressive. Dr. Cartwright says the weather in August presented each day, "several hours at a time attended with a breathless state of the atmosphere."

This summer yellow fever made its appearance quite early in New Orleans: many cases occurred among the ships' crews and others about the wharves as early as July; and it became epidemic about the first week in August. The arrival of steamboats from New Orleans was a thing of daily occurrence before the epidemic put a check to all kinds of business.

As soon as the disease was pronounced epidemic in Natchez this summer, as usual every family who had the means of escape, left the city. A few days afterwards, when the disease began to rage, ten or twelve families, including about sixty souls, removed to a cross-roads near Washington, known as Coonville, and there erected sheds and huts for a temporary residence. Being of the poorer class, they brought with them their beds, clothing, and all their movables, which were crowded into their small, ill-ventilated apartments. Here they remained in apparent security for a week or ten days, when cases of yellow fever began to develope among them. In a few days it spread among them with great mortality, and in a few weeks there were about twenty deaths among them, besides as many recoveries. The disease assumed its most malignant character here, and was precisely the same disease that prevailed in Natchez, and was equally fatal. At this time the whole country was as healthy as usual, and no disease of the kind existed any where, except where the infection could be traced to Natchez. However, at length the air of the encampment at Coonville became infected, and was avoided by persons in the vicinity as carefully as the air of Natchez itself.

But in the meantime five persons who had visited Coonville, and had been exposed to no other source of infection, contracted the disease and died in the country. It was admitted that the air of Coonville was as strongly infected as that of Natchez itself.

In this case the infected air of Natchez in the form of *fomites*, contained in beds, bedding, clothes, &c. was carried out and confined in these crowded, ill-ventilated wooden huts, until it took on the assimilating process, and produced an epidemic, which would have spread as readily through five thousand persons if they had been congregated there. No one ever presumed to ascribe this little epidemic to city filth, grave-yards, or putrid bacon.

Dr. Cartwright, in a lengthy essay published in the Medical Recorder, ascribed the epidemic in Natchez this year to a lot of putrescent bacon in the south-west part of the city, aided by other putrescent animal matters supposed to have existed about the city.* As to the bacon, I have long since been assured by Col. Fleming Wood, the alleged proprietor of the bacon, that the information on that subject was entirely erroneous; and that no such lot of bacon was in the city. Scarcely any one is sufficiently credulous, or so biassed by theory, as to believe that Natchez, which is known to be one of the most cleanly cities in the South, should be so infested with carrion, and other putrid matters, as are supposed to be necessary to bring on an epidemic. If any stranger were credulous enough to entertain such a belief, he would at once repudiate the idea when he should learn that Natchez has long been rather remarkable for the number of half-starved, rapacious vultures, which hover round in anxious expectation of such matters, which they devour in less than half the time required for the same performance by northern carrion-crows.

In 1824, the city was free from an epidemic.

*Epidemic of* 1825.—This epidemic began under the hill, or at the landing, among the clerks of a commission house and

* Medical Recorder, Vol. ix: pp. 5—7.

others near the steamboat landing. Cases began to multiply about the 20th of August. Several persons attacked were removed by their friends into the upper city, where they finally died. On the last of August the alarm was such, that a general flight of the citizens to the country ensued. The disease prevailed nearly two weeks near the landing before it began to spread in the upper city; and the first cases in the latter were easily traced to intercourse with the landing.

The disease continued to prevail until checked by a freeze on the first of November. The whole number of deaths in Natchez was about 150 souls. As usual, several deaths occurred late in November in persons who had left the city early, and returned after frost into their houses, which had remained closed.

This year the disease was carried to Washington. This is a pleasant, elevated, and cleanly village, six miles east of Natchez, containing about 100 hundred houses thinly distributed over a space half as large as Natchez. It had heretofore been the retreat of the merchants, mechanics, and others who wished to continue their trade with the country. This fall Washington was crowded with people, goods, and all the movables necessary for business and house-keeping. Nearly half the mercantile establishments carried their goods along, and every day for ten days brought out new supplies from Natchez and New Orleans. No unusual sickness prevailed in Washington or in the surrounding country. During the first twenty days of September, the mercury ranged as high 90° to 93° each day, and the air was sultry and oppressive.

During the first ten days after the arrival of the Natchez people, there were about 8 deaths from yellow fever in those who had contracted the disease before they left Natchez. About this time others who were citizens of Washington and who had not been in Natchez, began to sicken with yellow fever; and by the 18th of September the disease was considered epidemic in Washington, when nearly all the population fled.

The whole number of deaths in the Washington epidemic was about 60 souls. About ten cases and five deaths occurred among those who returned into houses which had been closed, and were not entirely disinfected by the cold weather.

Previous to this summer, Washington was known as a healthy retreat to the people of Natchez during their epidemic visitations, where the disease had never been known to spread. No one pretended to ascribe this epidemic to filth, or the usual sources to which it is ascribed in cities. Washington is remarkable for the absence of every thing like city filth.

Dr. Cartwright, however, did ascribe it to a quantity of "putrescent bacon," which he alleges to have been carried out from Natchez. Even this would be an admission that the disease was carried from Natchez to Washington. But the fact is, there was no such "putrescent bacon" as that to which he refers. The writer was any eye-witness to the whole of this epidemic, and resided within one hundred yards of the alleged putrid bacon, and he does not hesitate to say that the Doctor was misinformed on that subject.

I hesitate not to declare it as my belief, that there would have been no epidemic in Washington, unless the people and goods from Natchez had been crowded into it. Besides it must be remembered the merchants received large supplies of fall goods from New Orleans while they were in Washington, and among these were 8 or 10 bales of blankets to meet the annual fall demand of the planters.

The first citizens of Washington attacked were my patients; and their infection could be traced clearly to opening and handling those bales of blankets, and other goods brought from New Orleans and Natchez. The disease was imported to Washington as surely as it was to Coonville in 1823.

The intercourse between Natchez and New Orleans was uninterrupted: goods were almost daily received at Washington on the same day they were landed at Natchez.

Dr. Cartwright ascribed the epidemic this year, to causes similar to those which he adduced in 1823; to which he added "spoiled porter," "rotten sour-crout," and also a boat with some corn in it sunk near the wharf.

Dr. Merrill ascribed it to "loose earth" exposed to the sun, in making and filling up a wharf, and in levelling the streets. Others ascribed it to such combination of circumstances or causes, as their fancy might suggest. These causes had no agency whatever in producing the epidemic; and we have shown already that such causes are harmless as generators of yellow fever miasm. We assert without fear of successful refutation, that whenever these circumstances have synchronised with a yellow fever epidemic, they were only incidental occurrences, and not in any manner essential to its existence. It is certain that several epidemics have appeared in Natchez since 1825, without either of the causes assigned by Dr. Cartwright or Dr. Merrill.

It has been urged against the doctrine of importation, that in 1823 and 1825, previous to the visitation of the epidemics, there was a regular quarantine against boats from New Orleans. I admit there was a mock quarantine, but none that was effectual. Each boat from New Orleans at that time was required to round to at the quarantine ground, and permit the health officer to inspect her, when she passed on up to the landing at Natchez. It is well known, that although boats were subjected to this formality, no one was ever prohibited from discharging either freight or passengers. There never was a quarantine at Natchez, *on proper principles*, until the summer of 1841, and that quarantine excluded the disease from Natchez, although it was carried on to Vicksburg. We will speak of this again.

The next epidemic visitation in Natchez was in 1829. This was the mildest epidemic ever known in the city. The city was deserted early and the disease did not spread extensively.

For several years the city of Natchez escaped the visitation of yellow fever until the fall of 1837, when it again made its appearance.

*The epidemic of* 1837.—This began with a few cases called sporadic, about the 8th and 10th of September; and by the 15th it was considered epidemic. Many of the physicians denied the existence of yellow fever in the city until several cases terminated fatally with the genuine black-vomit, which none could dispute. The disease continued to spread gradually and with occasional abatements, until checked by frost about the 25th of November. The number of deaths from this epidemic was about 280, including hospital cases, many of which had been landed in a moribund state from steamboats direct from New Orleans. The epidemic this year was more mild and slow in its advances than usual, until the middle of October, when it began to rage with great malignity. This season the first cases in September frequently assumed some of the symptoms so mild that it was declared by some to be *bilious fever.*

The city of Natchez was as healthy as usual until the cases began to multiply; and it must be remembered that there were many cases of yellow fever landed from steamboats direct from New Orleans for ten days before any cases appeared among the residents of the city. The Natchez Hospital had been opened for the reception of indigent boatmen and others; and scarcely a boat passed up from New Orleans at this season of the year, that did not leave some yellow-fever patient at the hospital. It is a well-known fact on the lower Mississippi, that from the time yellow fever begins to occur in New Orleans, almost every boat that passes up leaves one or more yellow-fever patients at Natchez to be removed to the hospital, in passing to which they are carried through the most populous part of the city.

For several years previous to 1837 the Natchez Hospital had been closed against the reception of sick from the boats; and during this time there was no epidemic. But, a year before, the legislature had made provision for throwing open the hospital to the indigent sick; and it was in full operation when the yellow fever broke out in New Orleans, and scarcely a day passed without the reception of one or more patients from ascending boats, after the first of June; and after the first of August nearly all these were yellow-fever cases.

It may be worthy of notice here, that in the summer of 1841, when the quarantine was adopted, the hospital was closed, and no patients permitted to land from the boats, unless at private houses. As a general remark, there has never been yellow fever in Natchez unless when the hospital was open for the reception of indigent boatmen and others from the river. When the hospital is open, it is an inducement for all the steamboats passing up to land at Natchez, if for no other purpose than to relieve themselves of the sick, whom they might have to bury on their way above.

### YELLOW FEVER IS NOT ENDEMIC IN THE UNITED STATES.

By this I mean to say, that *yellow fever is a specific disease*, having no perfect analogy, or any characteristic symptoms which identify it with bilious fever, which is endemic in the United States.

The circumstances under which these two forms of disease prevail are widely different; the persons liable to each are different; the times and places where each prevails are different; the symptoms and pathology of each are different; and the impression left upon the system after recovery, is different; a mild case of yellow fever is not a bilious fever; and a severe and malignant case of bilious fever is not yellow fever.

The following are some of the principal points of difference between the two diseases—viz:

1. *Bilious fever* prevails in the south, chiefly in hot and showery weather, and more commonly in May, June and July; but sometimes in August and September. *Yellow fever* in the United States, never prevails as an epidemic until late in July, and generally, not before the middle of August or September; and always after a hot, dry, and sultry state of the weather.

2. *Bilious fever*, at times, prevails over certain districts of country, and generally among a sparse population, and is more common in country situations than in towns or cities. *Yellow fever* is confined as an epidemic, exclusively to towns and cities, and appears *first* in commercial ports and large trading towns, and spreads only among a dense population, in an impure air.

3. When *bilious fever* prevails, it is caused by a *hot and moist* atmosphere, producing a degree of insalubrity extending over many miles of surface continuously, and without any circumscribed limits. *Yellow fever* prevails, or becomes epidemic in *hot and dry* seasons, and is restricted to certain limits, and to a local atmosphere as clearly defined as the bounds of a town or village, or the limits of one or more streets in a city; or one or more houses, or even to a single ship; while all the exterior air is perfectly salubrious, and the country population is uncommonly healthy, being often exempt from any general disease.

4. *Bilious fever* exists in all grades, from the simplest remittent to malignant bilious fever, terminating fatally, and yet preserves its distinct character, never assuming any of the pathognomic symptoms of yellow fever. *Yellow fever* in like manner exists in all grades from the mildest to the most malignant form, without losing its distinct and pathognomic character, whether it terminates in convalescence or death.

5. Persons who are natives of tropical climates, or those who have become acclimated to yellow-fever malaria, or who have recovered from one attack, are mostly exempt from a subsequent attack of yellow fever, but may have repeated attacks of bilious fever. Those who have been subjected to frequent, or annual attacks of *bilious fever*, are more liable to subsequent attacks, than those who have never suffered from it; and they are equally as obnoxious to epidemic yellow fever, as those who have never had bilious fever.

6. *Bilious fever* occurs in an atmosphere but slightly, if at all, contaminated by respiration; yellow fever, as an epidemic, depends for its existence upon air contaminated by human respiration.

Observant practitioners of the South, who have been familiar with yellow fever and also with bilious fever, do not pretend to identify the two forms of disease. They know by experience and repeated observation, that they are essentially different forms of disease; that yellow fever is a disease *su i generis;* and that yellow fever and bilious fever do not prevail in the same local atmosphere. When bilious fever prevails generally in the surrounding country, the cities have no cause to apprehend epidemic yellow fever.

In this I am sustained by the testimony of all southern experience untrammelled by theory. The venerable Dr. Samuel Hogg, of Nashville, who practiced several years in Natchez, has assured me repeatedly of his firm conviction, that yellow fever is a specific disease, and partakes no more of the character of bilious fever, than of any other form of disease. In relation to the epidemic of 1837 in Natchez, he thus expresses himself in the Western Journal of Medicine and Surgery:*—"It was not, as has been supposed, a high or malignant grade of bilious fever; the whole surrounding country being more exempt from that disease than usual." . . . . "Also in most cases of recovery, the first satisfactory evidence of amendment, was a plentiful secretion of dark acrid bile."

After showing that it differed from all the ordinary diseases of the country, he remarks:—"If it were neither of the foregoing diseases, the question arises, *What was it?* I must briefly answer, that it was an assemblage of symptoms, or rational signs, indicating the modified condition of such organs as previous predisposing causes might have made obnoxious to the morbid influence of a specific cause, not cognizable to our senses—a poison *sui generis*, infecting a certain atmosphere."

On the 20th of October, 1839, the writer had an interview with the same distinguished individual, who had just returned from Nashville, by land, and through the interior of Mississippi, from north to south. The Doctor declared that in his route, he had passed leisurely through the whole length of the State, visiting most of the towns and villages on the way; and that while he daily heard of the ravages of yellow fever in

* See June No. for 1840—Hogg on the epidemic fevers of Natchez.

most of the cities and towns in the southwestern portion of the State, he was astonished to find so universal an exemption from disease of every kind, in the interior; and that he had not seen or heard of a case of bilious fever in the whole route. He then declared that the evidence was conclusive, that yellow fever and bilious fever were two separate and distinct diseases. He expressed his belief, that no discriminating observer, who had witnessed the facts of this summer, would contend otherwise; for the case presented by the summer and autumn of 1839, would amply convince any man of sound judgment. The writer has witnessed the same thing several times in the lapse of twenty years. From the experience of twenty years' practice in the vicinity of Natchez, I have learnt that a hot and dry season during the summer months is a certain guaranty of health; a hot and wet season is a sure concomitant of sickness, and especially of bilious and congestive fevers. As a general rule it is known proverbially to be healthy in the country while yellow fever is raging in the cities and river towns. The inference is conclusive, that ordinary bilious and remittent fevers originate from causes, and under circumstances, entirely different from those which lead on yellow fever in the cities. So far as analogical reasoning can convince, these facts prove clearly that yellow fever is a disease *sui generis;* besides which we see that the symptoms, the accession, the course, the duration, and the termination, are all different in the two diseases. The organs and tissues mainly implicated are different.

Yellow fever is properly a disease of tropical climates. Like some tropical plants it may be transported to a more northern climate, and for a time may flourish; but the frost of our winters will utterly destroy it, and it must be annually renewed. Without this annual transplanting or renewal, in our climate it would die and become extinct. But the constant commercial intercourse with the tropical ports, serves to keep up the annual supply of this exotic. It can be successfully transplanted in our climate, only for a short time, when the weather in certain sections of the country is strongly assimilated to the climate of the West Indies—such as in the island of Curacoa.

To establish the diversity between yellow fever and bilious fever, I am extremely happy to be able to cite the authority of Professor Caldwell,* one of the most distinguished teachers of medicine in the United States. He supposes the poison of yellow fever may be constituted of different proportions of the same elementary principles, which enter into the morbific miasm of bilious fever; but that the proportions of these constituents are so varied, that the poison resulting is entirely different in its effects upon the human system. He remarks, that, "wherever genuine yellow fever reigns as an epidemic, it reigns alone." Speaking of the peculiar nature of yellow fever, he continues: "Throughout the whole range its type is the same. It continues to be every where yellow fever; although in different places of very different grades. Under its lightest and simplest modification it is less severe, and more easily cured, than intermitting fever. Yet in type and character it is as different from that disease now, as when marked by its highest grade of malignity. Its strength and power to injure are gone; but in its form it has suffered no mutation."

In illustration of the diversity of the diseases, I will state the following fact which fell under my notice. A friend of mine had fifteen negro slaves in New Orleans, where they had become thoroughly acclimated to yellow fever infection, and were exempt from its attacks during several epidemics in the city. In the spring of 1835, he removed them to a plantation on the Mississippi river a few miles above Natchez, and about 300 miles above New Orleans. During the following summer bilious fever prevailed to a considerable extent through the country, the weather being hot and showery. During the season every one of these negroes had severe attacks of bilious fever, in its severest grade, and one of them died from it who had been raised in New Orleans, and was not liable to yellow

* Medical and Physiological Memoirs—Prize Questions, pp. 210—211, &c.

fever. The others were removed to New Orleans in 1838, and remained there during the most malignant epidemic of 1839, in the most perfect health. This principle might be illustrated in a hundred cases. But it appears to me unnecessary to enter further into a discussion of the question whether yellow fever be a disease *sui generis*, or only a high grade of bilious fever.

I have been told by enlightened physicians and teachers of medicine in the region of the Ohio, that the two diseases are identical; that the bilious fevers on the Ohio frequently run into the yellow-fever grade; and that they have seen genuine yellow fever in the Ohio valley, of local origin. I do not hesitate to declare it as my opinion, that they are altogether mistaken on that point. A very strong argument in my favor is the fact, that scarcely a year passes without many young physicians emigrating to the south, as fully imbued with those doctrines as their learned teachers could desire; and they never do recognize yellow fever when they first meet with it; and almost invariably it is a source of much mortification to them afterwards. The older practitioners who are familiar with it, recognize it at once, and in any stage; those who have never seen it, but who are confident of their judgment, almost invariably deny the existence of yellow fever until it has made havoc among their patients and derided their skill. The medical faculty of Vicksburg in 1841, denied the existence of yellow fever in their city for two weeks after it was sweeping them off at the rate of 8 or 10 a day. This was the case too after several of them, unconsciously, had seen cases in the fall of 1839. In both cases it was called congestive fever. Our most eminent physicians, on their first introduction to the disease, were placed in the same unpleasant predicament. An intelligent physician of Vicksburg, informed me in December 1841, that for two weeks they had no suspicion of yellow fever, and considered the epidemic as some new form of congestive fever; and of course the treatment was not such as it would have been under a certain knowledge of its true character.

Along with the few advantages which have resulted from the dissemination of the doctrines of Dr. Rush, relative to the local origin of yellow fever, it must be admitted that several important evils have come. Of these, not the least is the utter disregard of many prudential measures, calculated to prevent the spread of the pestilence, as well as its introduction into our sea-ports. His doctrines inculcate an imprudent exposure of the healthy to the sources of disease, to infected atmosphere; and thereby to unnecessarily endanger the lives of our citizens, and the prosperity of our most flourishing cities. This exposure is inculcated under a vague conception of the terms, contagion and infection, at least, by those who are thus induced to become its victims. Relying upon some indefinite meaning of the terms, and utterly ignorant of the true laws which govern the origin and spread of yellow fever in the south-west, many enterprising and generous young men from the north, have been annually sacrificed to the prejudices of education. Hundreds every year fall victims, who. under proper views of this disease, might pursue the path of safety.

(TO BE CONCLUDED.)

